# Peroxisome Proliferator-Activated Receptor-γ Is a Potent Target for Prevention and Treatment in Human Prostate and Testicular Cancer

**DOI:** 10.1155/2008/249849

**Published:** 2008-02-19

**Authors:** Masahide Matsuyama, Rikio Yoshimura

**Affiliations:** Department of Urology, Osaka City University Graduate School of Medicine, 1-4-3 Asahi-machi, Abeno-ku, Osaka 545-8585, Japan

## Abstract

Peroxisome proliferator-activated receptor-γ (PPAR)-γ is a ligand-activated transcriptional factor belonging to steroid receptor superfamily. PPAR-γ plays a role in both adipocyte differentiation and tumorigenesis. Up to date, PPAR-γ is expressed in various cancer tissues, and PPAR-γ ligand induces growth arrest of these cancer cells. In this study, we examined the expression of PPAR-γ in prostate cancer (PC) and testicular cancer (TC) by RT-PCR and immunohistochemistry, and we also examined the effect of PPAR-γ ligand in these cells by MTT assay, hoechest staining, and flow cytometry. PPAR-γ expression was significantly more extensive and intense in malignant tissues than in normal tissues. PPAR-γ ligand induced the reduction of malignant cell viability through apoptosis. These results demonstrated that the generated PPAR-γ in PC and TC cells might play an important role in the tumorigenesis. PPAR-γ may become a new target in the treatment of PC and TC.

## 1. INTRODUCTION

Prostate cancer (PC) comprises 32% of all cancers in American men and is on the increase worldwide. Because of increased screening, PC is frequently diagnosed at a clinically localized stage, making it amenable to the therapy. Nevertheless, it remains the second most common cause of cancer death in men. These patients generally respond to androgen deprivation therapy, but the vast majority eventually experience disease progression and become refractory to sustained hormonal manipulation. Typically, such patients progress with a rise in their serum prostate-specific antigen level. Unfortunately, standard therapeutic options at this stage of disease are limited, and while there has been some success with chemotherapy for hormone-refractory prostate cancer patients, the response is generally short lived [1].

Testicular cancer (TC) is very rare with over 90% of all TC being germ cell tumors (seminoma and nonseminoma), and the remaining percentage nongerminal tumors. The survival rate of TC has improved in recent years, reflecting the development and refinement of effective combination chemotherapy. However, it is still necessary to improve the treatment of TC.

Angiogenetic factors play an important role in prostate and testis as in other organs [2], and although various potential angiogenetic factors have been identified in PC and TC, it is still unclear by which process PC and TC cells become angiogenic. Thus, the challenge is to discover new treatment strategies that target androgen-independent PC and TC. The identification of molecular targets involved in the tumorigenesis and progression of PC and TC provide opportunities for the development of new agents with greater therapeutic potential and better specificity. Patients with advanced or recurrent disease are suitable candidates for studies that test the efficacy of these new agents.

Peroxisome proliferator-activated receptors (PPARs) are lipid-activated transcription factors that function as important regulars of lipid and glucose metabolism, adipocyte differentiation, and energy homeostasis. PPAR subtypes (α, β, and γ) have been found. Both PPAR-α and -γ mediate the action of the hypolipidemic fibrates and antidiabetic thiazolidinediones. PPARs therefore play a role in metabolic conditions such as dyslipidemia and type 2 diabetes, leading to atherosclerosis development [3]. PPARs also have regulatory role in inflammation.

PPAR-γ provides a strong link between lipid metabolism and regulation of gene transcription [4]. PPAR-γ acts in adipose tissue and promotes lipogenesis under anabolic conditions. Recently, the receptor has also been implicated in inflammation and tumorigenesis. Significant evidence from many experimental systems suggests that PPAR-γ is important in carcinogenesis.

PPAR-γ is up regulated in malignant tissue, and PPAR-γ ligands induce terminal differentiation in human breast and colon cancer cells [5, 6], and inhibit the growth of human lung and gastric cancer cells [7, 8]. In addition, PPAR-γ ligands induce growth arrest through apoptosis in macropharge, fibrobrasts, and endothelial cells [3, 9, 10]. Our research elucidates the expression of PPARs in urological cancers and administration of PPAR-γ ligands as an anticancer therapy [11–15]. Several reports support the expression of PPAR-γ and the efficacy of PPAR-γ ligands in PC [16–18]. However, no further data on TC and PPAR-γ have been documented in other reports.

Our research focuses on the relationship between PPAR-γ and male reproductive system (prostate and testis) and on the anticancer effect of PPAR-γ ligands.

## 2. METHODS

### 2.1. Tumor specimens

Prostate specimens were obtained from 156 patients with PC; 15 with prostatic intraepithelial neoplasia (PIN); 20 with benign prostatic hyperplasia (BPH), who underwent biopsy due to serum prostate-specific antigen increase; and 12 patients with normal prostate (NP) tissues who underwent total cystectomy due to bladder cancer.

Testis specimens were obtained from 72 TC patients, and from 20 NT patients who underwent orchiectomy for PC. Tumor tissues, nontumor tissues, vascular endothelium, and interstitial tissues from the subjects were preserved in 10% formalin and embedded in paraffin, serially sectioned onto microscope slides at a thickness of 4 μm.

### 2.2. Antibodies

PPAR-α, -β, and -γ are affinity-purified goat polyclonal antibodies. We purchased these antibodies from Santa Cruz Biotechnology Inc (Santa Cruz, Calif, USA). They demonstrated about the source of these antibodies, PPAR-α and -γ are affinity-purified goat polyclonal antibodies raised against a peptide mapping at the amino terminus of PPAR-α and -γ of human origin (α differs from corresponding mouse sequence by amino acids; γ is identical to the corresponding mouse sequence). PPAR-β is an affinity-purified goat polyclonal antibody raised against a peptide mapping at the carboxy terminus of PPAR-β of human origin (differs from corresponding mouse sequence by two amino acids). About specificity of these antibodies, PPAR-α and -β react with those of mouse, rat and human origin by Western blotting and immunohistochemistry. PPAR-γ also reacts with PPAR-γ1 and PPAR-γ2 of mouse, rat, and human origin by Western blotting and immunohistochemistry. These specific antibodies do not cross-match either each other, nor do they cross-react with each other.

### 2.3. RT-PCR

Total RNA was isolated from PC tissues, BPH and NP tissues (fresh tissues) by guanidium thiocyanate-phenol-chloroform method. We performed an RT-PCR procedure to determine the PPAR-α, -β, and -γ mRNA expression as described previously [19]. In short, total RNA was used as a template for DNA synthesis using a superscript preamplification system (GIBCO-BRL) according to the manufacturer instructions. PCR was performed with each cDNA; PPAR-α, -β, and -γ; or G3PDH primer and Taq DNA polymerase (NIPPON GENE, Toyama, Japan). The synthetic oligonucleotides were obtained from Nippon Flour Mills (Kanazawa, Japan). We used G3PDH mRNA as a control.

The primers used were as follows:

- PPAR-α: sense; $5^{\prime }$-CCAGTATTTAGGACGCTGTCC-$3^{\prime }$ and antisense $5^{\prime }$-AAGTTCTTCAAGTAGGCCAGC-$3^{\prime }$;
- PPAR-β: sense; $5^{\prime }$-AACTGCAGATGGGCTGTAAC-$3^{\prime }$ and antisense $5^{\prime }$-GTCTCGATGTCGTGGATCAC-$3^{\prime }$;
- PPAR-γ: sense; $5^{\prime }$-TCTCTCCGTAATGGAAGACC-$3^{\prime }$ and antisense $5^{\prime }$-GCATTATGAGACATCCCCAC-$3^{\prime }$;
- human G3PDH: sense; $5^{\prime }$-CCACCCATGGCAAATT CCATGGCA-$3^{\prime }$ and antisense; $5^{\prime }$-TCTAGAGGGCAG GTCAGGTCCACC-$3^{\prime }$.

The primer sets yield PCR products of 492, 484, 474, and 598 base pair for PPAR-α, -β, and -γ or G3PDH, respectively. Reactions were incubated in an automatic heat-block for 30 cycles of denaturation 40 seconds, $94^{{}^{\circ}}$C ; annealing for 50 seconds, $50^{{}^{\circ}}$C; extension for 50 seconds, $72^{{}^{\circ}}$C [19]. PCR products were run on 2% agarose gel in TAE buffer (40 mM Tris acetate, 1 mM EDTA) and visualized by ethidium bromide staining.

### 2.4. Immunohistochemistry

Tissues sections (4 μm thick) were incubated with anti-PPAR-α, -β, and -γ antibody (2 μg/mL) or purified normal goat IgG (2 μg/mL) in a humid chamber for 24 hours, and further incubated with biotinylated rabbit antigoat IgG (Vector Laboratories, Inc. Burlingame, Calif, USA) for 30 minutes. After washing with PBS, the sections were incubated with the vectastatin avidin-biotin peroxidase complex kit (Vector, Burlingame, Calif, USA) [20] for 45 minutes. Color was developed by immersing the sections in a solutions of 0.05% wt/vol 3,$3^{\prime }$-diaminobenzidine tetrahydrochloride (Sigma-Aldrich, St. Louis, Mo, USA). The sections were counterstained with hematoxylin (Sigma-Aldrich, St. Louis, Mo, USA).

### 2.5. Statistical analysis

The extent and intensity of staining with PPAR-α, -β, and -γ antibodies were graded on a scale of 0 to 4 (+) by two blind observers on two separate occasions using coded slides, and an average score was calculated [21]. Staining was classified into 5 grades from 0 to 4 (+) according to the intensity of staining and the number of positive cells. The observers assessed all tissues on the slides to assign the score. A 4 (+) grade implies that all staining was maximally intense throughout the specimen, whereas 0 implies that staining was absent throughout the specimen. The microanatomical sites of staining were also recorded. To quantify the expressions of PPAR-α, -β, and -γ, the same two pathologists made assessments throughout the study, staining control specimens simultaneously. This method, therefore, increases the credibility of data. In addition, all specimens were reassessed, which also contributed to the exclusion of any subjective variability.

### 2.6. Cell cultures

The human PC cell lines (LNCaP, PC3, DU-145) and TC cell line (NEC-8) were obtained from Health Science Research Resources Bank (Osaka, Japan). Cells were grown in culture flask (Nunc, Roskilde, Denmark) in RPMI 1640 supplemented with 10% FBS, 100 U/mL of penicillin and 100 μg/mL of streptomycin in a humidified 5% CO_2_ atmosphere at $37^{{}^{\circ}}$C. The media were changed every 3 days and the cells were separated via trypsinization, using trypsin/EDTA when they reached subconfluence.

### 2.7. Cell proliferative studies

Troglitazone (thiazolidinedione compounds) was obtained from Sankyo Pharmaceuticals (Tokyo, Japan). 15-deoxy-$\Delta^{12,14}$-prostaglandin J_2_ (15-d-PGJ_2_) was purchased from Cayman Chemical Company (St. Louis, Mo, USA). GW9662 was purchased from BIOMOL Research Laboratories Inc. (Pa, USA). Approximately $1.0\times 10^{4}$ cells (all PC and TC cell lines) placed onto 8×8 mm diameter multichamber slides (Nunc, Copenhagen, Denmark) were treated with troglitazone and 15-d-PGJ_2_ (5–40 μM) dissolved in ethanol. The final concentration of ethanol was <0.05%. Cell viability was measured after 48 hours by a microplate reader using a modified 3-[4, 5-dimethylthiazol-2-thiazolyl]-2,5-diphenyltetrazolium bromide (MTT) assay (WST-1 assay; Dojindo, Kumamoto, Japan) and presented as the percentage of control-culture conditions (N=6).

### 2.8. Flow cytometry (annexin V and propidium iodide staining)

The effects of PPAR-γ ligands on PC (PC3) and TC (NEC-8) cell lines were determined by dual staining with Annexin V-FITC and propidium iodide using Annexin V-FITC Apoptosis Detection Kit I (Biosciences Pharmingen, Calif, USA). Annexin V-FITC and propidium iodide (PI) were added to the cellular suspension as in the manufacturer instruction, and sample fluorescence of $1.0\times 10^{4}$ cells was analyzed flow cytometry. Flow cytometry was with FACScan (Becton Dickinson, Heidelberg, Germany). Cell which were Annexin V-FITC positive and PI negative were identified as early apoptosis. Cell which were Annexin V-FITC positive and PI positive were identified as late apoptosis or necrosis.

### 2.9. Flow cytometry (identification of DNA fragmentation)

The assay was performed by TdT-mediated dUTP Nick End Labelling (TUNEL) method using APO-DIRECT kit (Becton Dickinson). Following the experiments, PC (PC3) and TC (NEC-8) cell lines in suspension ($1\times 10^{6}$/mL) were fixed with 1% PBS, washed in PBS, and suspended in 70% (v/v) ice-cold ethanol. The cells were stored in ethanol at $-20^{{}^{\circ}}$C until use. The positive and negative controls and the sample were stained with FITC-dUTP by incubation in terminal deoxynucleotidyl transferase buffer as in the manufacturer instruction, and sample fluorescence of $1\times 10^{4}$ cells was analyzed by flow cytometry (Becton Dickinson). Results are given as % of TUNEL-positive cells.

### 2.10. Detection of apoptosis by Hoechst staining

DNA chromatin morphology was assessed using Hoechst staining. PC (PC3) and TC (NEC-8) cell ($5\times 10^{5}$ cells) were incubated with 20 μM PPAR-γ ligands for 24 hours. Cells were washed by RPMI-1640 and labeled with 8 mg/mL of hoechest 33342 (Sigma-Aldrich Japan K.K. Tokyo, Japan) for 10 minutes; PI (Sigma-Aldrich Japan K.K. Tokyo, Japan) was added (10 mg/mL final concentration), and the cells were examined by fluorescence microscopy.

### 2.11. Statistical analysis

All results are presented as the mean ± SD. Analysis of data was performed using the analysis of variance (ANOVA) [22].

## 3. RESULTS

### 3.1. Tumor specimens

#### 3.1.1. PC tissue sample

The 156 patients with PC were male aged 59–78 years (mean age 67±5.3 years). We used Gleason score to evaluate PC. Gleason score is given to PC based upon its microscopic appearance. Gleason score is important because higher Gleason scores are associated with worse prognosis. This is because higher Gleason scores are given to cancer which is more aggressive. Gleason score ranges from 2 to 10. Gleason score of 2 is associated with the best prognosis and a score of 10 with the worst. The final score is a combination of two different scores which each range from 1 to 5. Gleason score is as follows: low group: Gleason score, 2, 3, 4, 5, middle group: Gleason score, 6, 7, 8, high group: Gleason score, 9, 10. In clinical PC, Gleason score is almost over 5.

The 50 patients were in the low group, 54 were in the middle group, and 52 were in the high group. The 15 patients with PIN averaged 64±5.9 (52–73) years. The 20 patients with BPH averaged 68±4.7 years (59–75), and all had nodular hyperplasia. The 12 patients with NP averaged 52±7.6 (44–62) years.

#### 3.1.2. TC tissue sample

The 72 TC patients were a mean age of 31.0±12.3 years. Tumors of single histologic types were found in 58 patients and more than two histological types in 14 patients. Seminoma occurred in 31 patients, embryonal carcinoma in 8 patients, yolk sac tumor in 7 patients, choriocarcinoma in 7 patients, and teratoma in 5 patients. Tumors having more than two histologic types included embryonal carcinoma and teratoma in 4 patients, choriocarcinoma and other types in 3 patients, and other combinations in 7 patients. The average age of 20 patients NT tissues was 61.4±8.6 years.

### 3.2. RT-PCR

To check PPAR-α, -β, and -γ mRNA variation, RT-PCR was performed with total RNA extracted from all specimens. Using specific primers for PPAR-α, -β, and -γ and G3PDH, the amplification predicted, respectively, fragments of 492, 484, 474, and 598 base pair (bp) in length.

#### 3.2.1. PC tissue sample

**Figure 1:**
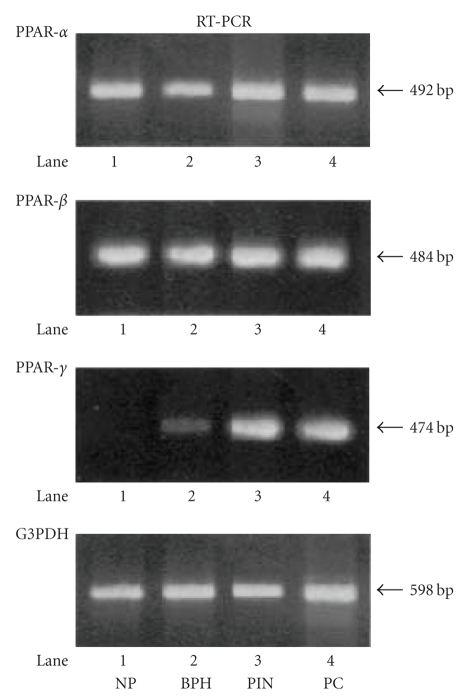
RT-PCR analysis of PPAR-α, -β, and -γ in prostate tissue samples from the patients with PC, PIN, BPH and NP. A slight, but clear, band of PPAR-α and -β m-RNA was detected in all samples. However, the specific band of PPAR-γ mRNA in the samples from prostate cancer (PC) and prostatic intraepithelial neoplasia (PIN) was detected, while samples from benign prostatic hyperplasia (BPH) displayed a very weak band, and in a sample from normal prostate (NP) no clear band was detected.

The PPAR-α and -β mRNA were detected in PC, PIN, BPH, and NP samples. However, we detected a specific band of PPAR-γ mRNA in the samples from PIN and PC, and we also detected a very weak specific band of PPAR-γ mRNA in the sample from BPH, whereas sample from NP displayed no band of PPAR-γ mRNA (see Figure 1).

#### 3.2.2. TC tissue sample

The PPAR-α and -β mRNA were detected in all TC and NT samples. However, we detected a specific band of PPAR-γ mRNA in all TC groups, whereas sample from NT displayed no band of PPAR-γ mRNA.

### 3.3. Immnohistostaining of PPAR-α, -β, and -γ

To assess the tissue distribution of PPAR-α, -β, and -γ polypeptides, we stained paraffin-embedded samples with the affinity-purified PPAR-α, -β, and -γ antibodies that recognize specifically PPAR-α, -β, and -γ. The specificity of this antibody was proved by the previous experiments [23].

#### 3.3.1. PC tissue sample

**Figure 2:**
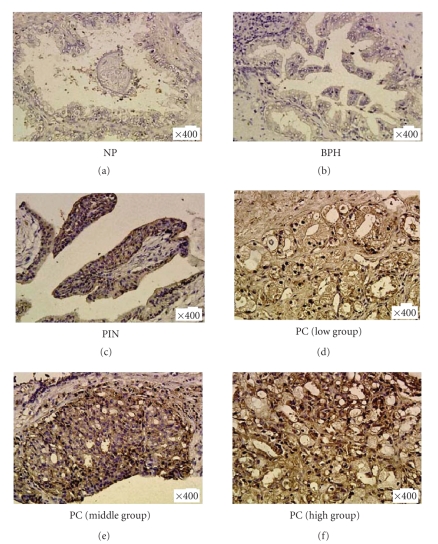
Representative immunostaining for PPAR-γ in prostate tissues samples. A significant strong PPAR-γ expression in all prostate cancer (PC) group tissues and prostatic intraepithelial neoplasia (PIN) tissue was detected, whereas PPAR-γ expression is very weak in benign prostatic hyperplasia (BPH) tissues and normal prostate (NP) tissue.

PPAR-α, and -β were expressed in PC, PIN, BPH, and NP tissues. Although very weak expression of PPAR-γ was found in BPH and NP tissues, PPAR-γ was strongly expressed in all PC groups and PIN (see Figure 2).

#### 3.3.2. TC tissue sample

**Figure 3:**
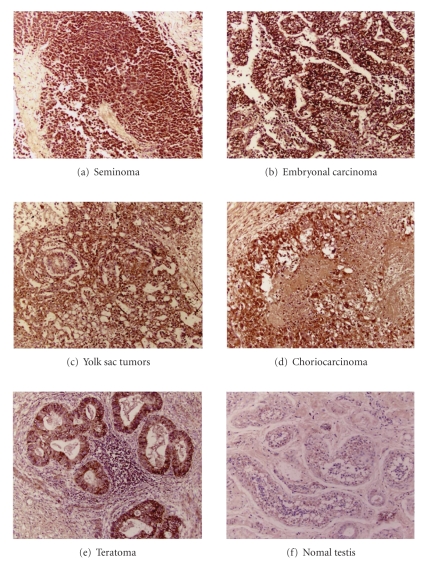
Representative immunostaining for PPAR-γ in testicular tissues samples. A significant strong PPAR-γ expression in all testicular cancer (TC) tissues was detected, whereas PPAR-γ expression is very weak in normal testis (NT) tissues.

PPAR-α, and -β were expressed in all TC and NT tissues. Although no expression of PPAR-γ was found in NT tissues, PPAR-γ was strongly expressed in all TC groups (see Figure 3).

### 3.4. Statistical analysis of PPAR-α, -β, and -γ immunostaining

To extent and intensity of staining with PPAR-α, -β, and -γ, antibody was graded 0 to 4 (+) by 2 blind observers.

#### 3.4.1. PC tissue sample

PPAR-α, -β immunostaining were significantly intense in all cases. There were no differences among PC, PIN, BPH, and NP. There was no significant difference of the intensity of PPAR-α, -β staining between PC, PIN, BPH, and NP. However, PPAR-γ immunostaining was significantly more extensive and intense in tumor cells (mean: low group; 2.6±0.7, middle group; 2.7±0.9, high group; 3.3±1.0, P<.01) and in PIN (mean: 2.5±0.8, P<.01) than in tissue of BPH (mean: 0.8±0.6). PPAR-γ staining was also high in blood vessels and stromal tissues of prostate cancer and PIN, with no significant difference between them (1.8–2.0). However, the expression of the PPAR-γ in the blood vessels and stromal tissues from BPH and NP was at the basic level (0.5–0.7) (see Table 1).

#### 3.4.2. TC tissue sample

PPAR-α, -β immunostaining were significantly intense in all TC groups and NT. However, PPAR-γ immunostaining was significantly more extensive and intense in tumor cells and blood vessels of the TC groups than in NT. There was no significant differences occurred between all TC group in tumor cells and blood vessels (see Table 2).

### 3.5. PPAR-γ ligands induced growth inhibition in PC and TC cell lines by MTT assay

To investigate the effects of PPAR-γ ligands on all PC (LNCaP, PC3, DU-145) and TC cell (NEC-8) lines proliferation, we analyzed cell viability in vitro by modified MTT assay.

#### 3.5.1. PC cell line

PPAR-γ ligands induced the reduction of cell viability with the half-maximal concentration of growth inhibition of all PC cell lines (LNCaP, PC3, DU-145) in the range of 5–40 μM (see Table 3). PPAR-γ ligands stopped the growth of all PC cell lines.

#### 3.5.2. TC cell line

Similar to PC cell lines, PPAR-γ ligands induced the reduction of cell viability with the half-maximal concentration of growth inhibition of TC cell line (NEC-8) in the range of 5–40 μM (see Table 3). PPAR-γ ligands stopped the growth of TC cell line (NEC-8).

### 3.6. PPAR-γ ligands induced apoptosis by flow cytometry

To evaluate whether or not cell death induced by PPAR-γ ligands was through apoptosis, we evaluated using flow cytometry. The higher left quadrants represent early apoptosis (Annexin V-FITC-positive cells and PI-negative cells). The higher right quadrants represent late apoptosis or necrosis (Annexin V-FITC-positive cells and PI-positive cells).

#### 3.6.1. PC cell line

**Figure 4:**
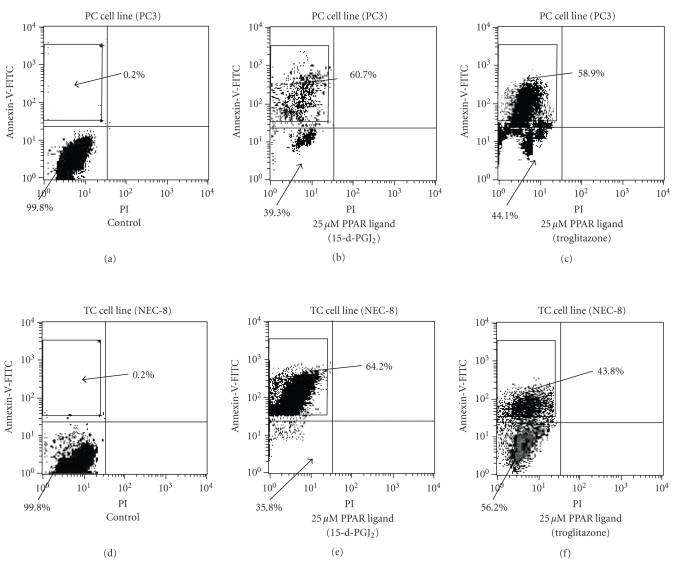
Effects of PPAR-γ ligands on apoptosis by flow cytometry in PC and TC cell lines. PC cells (PC3) and TC cell (NEC-8) lines with treatment of 25 μM 15-d-PGJ_2_ could induce early apoptosis not late apoptosis or necrosis. The higher left quadrants represent early apoptosis (Annexin V-FITC-positive cells and PI-negative cells). The higher right quadrants represent late apoptosis or necrosis (Annexin V-FITC-positive cells and PI-positive cells). Diagrams of FITC-Annexin V/PI flow cytometry are presented.

**Figure 5:**
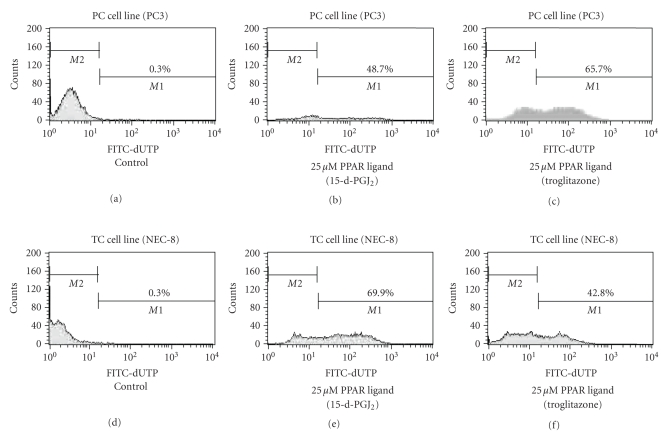
PPAR-γ ligands induce DNA fragmentation in PC and TC cell lines. 15-d-PGJ_2_ (2 μM) could induce DNA fragmentation in PC cell (PC3) and TC cell line (NEC-8). Typical flow cytometry analysis histograms are presented.

PC cell line (PC3) with treatment of 25 μM PPAR-γ ligand (15-d-PGJ_2_) could induce early apoptosis, not late apoptosis or necrosis (see Figure 4) and DNA fragmentation (see Figure 5). Diagrams of FITC-Annexin V/PI flow cytometry and typical flow cytometry analysis histogram are presented.

#### 3.6.2. TC cell line

TC cell line (NEC-8) with treatment of 25 μM PPAR-γ ligands (15-d-PGJ_2_) could induce early apoptosis not late apoptosis or necrosis (see Figure 4) and DNA fragmentation (see Figure 5). Diagrams of FITC-Annexin V/PI flow cytometry and typical flow cytometry analysis histogram are presented.

### 3.7. Effect of PPAR-γ ligands in induction of apoptosis on PC and TC cell lines

To evaluate whether or not cell death induced by PPAR-γ ligands was through apoptosis, we evaluated the chromatin morphology of PC (PC3) cell and TC cell (NEC-8) lines using hoechst staining.

#### 3.7.1. PC cell line

**Figure 6:**
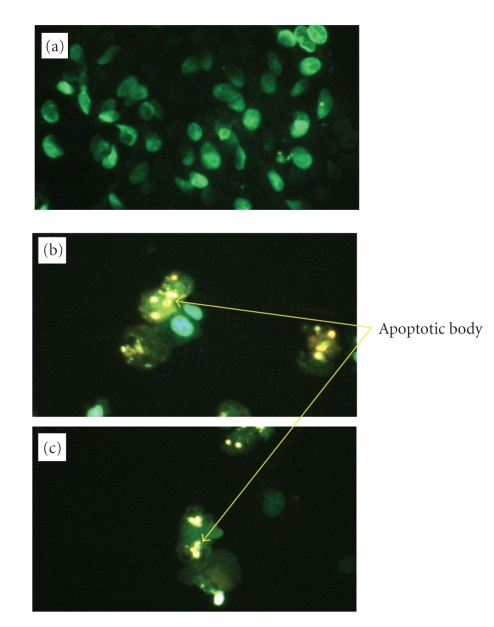
Effects of PPAR-γ ligands in induction of apoptosis on human PC cell line. PC cell line (PC3) treated with PPAR-γ ligands ((b); 20 μM Troglitazone, (c); 20 μM 15-d-PGJ_2_) showed significant chromatin condensation, cellular shrinkage, small membrane-bound bodies (apoptotic bodies), and cytoplasmic condensation. These cellular changes were typically redundant characteristics of apoptosis. PC cells without PPAR-γ ligands maintained normal chromatin patterns and cell size (a). Typical photographs are presented.

PC cell line (PC3) treated with PPAR-γ ligands showed significant chromatin condensation, cellular shrinkage, small membrane-bound bodies (apoptotic bodies), and cytoplasmic condensation. These cellular changes were typically redundant characteristics of apoptosis. PC cell lines (PC3) without PPAR-γ ligands maintained normal chromatin patterns and cell size (see Figure 6). Typical photographs are presented in Figure 6.

#### 3.7.2. TC cell line

Similar to PC cell line, TC cell line (NEC-8) treated with PPAR-γ ligands showed significant chromatin condensation, cellular shrinkage, small membrane-bound bodies (apoptotic bodies), and cytoplasmic condensation. These cellular changes were typically redundant characteristics of apoptosis. TC cell line without PPAR-γ ligands maintained normal chromatin patterns and cell size.

## 4. DISCUSSION

PPAR-α is highly expressed in the liver, heart, kidney, muscle, brown adipose tissue, and gut, which exhibit high carbolic rates of fatty acid. PPAR-β may be expressed ubiquitously and its function is relatively unknown. Recent studies suggest that PPAR-β may be a target for nonsteroidal antiinflammatory drugs (NSAIDs)-induced tumor suppression in colorectal tumors. PPAR-γ is expressed at high level in adipose tissue and is a critical regulator of adipocyte differentiation. In addition, PPAR-α, and -γ have been considered important immunomodulatory factors. PPAR-α knockout mice exhibit exacerbated inflammatory responses, and leukotriene B_4_, a chemotractic mediator, appears to regulate the clearance of itself as an agonist of PPAR-α. However, PPAR-γ is also expressed in the immune system, in the spleen monocytes, bone-marrow precursors, and helper T-cell clones. PPAR-γ is also expressed in chondrocytes as well as in synovial and bone tissues. Recent data have shown that PPAR-γ ligands lead to inhibition of phorbol ester-induced nitric oxide and macropharge-derived cytokines such as tumor necrosis factor-α (TNF-α), interleukin-1β (IL-1β) and interleukin-6 (IL-6), chemokines, and adhesion molecules, in part by antagonizing the activities of transcriptional factors [7].

Recently, it has been evidenced that thiazolidinedione, a new class of antidiabetic as a specific ligand for PPAR-γ, and retinoic receptor agonists can regulate differentiation of cancer cells [24], and that nuclear-acting prostanoids, including 15-d-PGJ_2_, are potent activators of the PPAR-γ receptor isoform [25, 26]. In fact, 15-d-PDJ_2_ induces apoptosis in macropharge, endothelial cell, choriocarcinoma cell [3, 10, 27], as well as thiazolidinediones-induced fibroblast apoptosis [9]. PPAR-γ ligands also inhibit vascular endothelial cell growth factor-induced angiogenesis in vivo [28]. Angiogenesis is important for tumorigenesis. Antiangiogenetic therapy is highly promising since it does not induce aquired anticancer drug resistance [29, 30]. Drevs et al. demonstrated the effect of PTK787/ZK 222584, a specific inhibitor of vascular endothelial growth factor receptor tyrosine kinases, on primary tumor, metastasis, vessel density, and blood flow in an animal model of renal cell carcinoma [31]. PPAR-γ agonists induce apoptosis in endothelial cells and inhibit vascular endothelial growth factor-induced angiogenesis in rats. Therefore, PPAR-γ ligands may have anticancer effects through inhibition of cell proliferation and angiogenesis.

In this time, concerning about PC, we demonstrated a stronger expression of PPAR-γ in PC and PIN tissues than in BPH or NP tissues by immunohistochemical staining and RT-PCR. We classified 3 categories (epithelial cells, blood vessels, and stromal tissues) in PC, PIN, BPH, and NP tissues, and examined the intensity of PPAR-α, -β, and -γ expressions in all tissue categories. There were no significant differences in the intensity of PPAR-α and -β in PC, PIN, BPH, and NP tissues. However, in all categories, PPAR-γ expression was significantly more extensive and intense in PC and PIN tissues than in BPH and NP tissues, and PPAR-γ expression was higher in G3 cancer than in G1 cancer. Paltoo et al. demonstrated that there were no significant differences between PPAR-γ expression in grades and stages [16]. Using competitive PCR, these differences may be demonstrated in the near future.

Next, we demonstrated that PPAR-γ ligands induced reduction of the viability in PC cells in the range of 5–40 μM by using MTT assay. Furthermore, we also demonstrated that PC cells treated with PPAR-γ ligands could induce early apoptosis and DNA fragmentation in PC cells. Subbarayan et al. have also demonstrated similar results [17]. Several reports support the efficacy of PPAR-γ ligands in PC [16, 18]. We expect that additional research will be progressed.

Concerning about TC, we demonstrate stronger expression of PPAR-γ in all tissue types of TC than in normal testicular tissues by immunohistochemical staining and RT-PCR. There were no significant differences among 5 histopathologic groups. We classified 2 categories (epithelial cells and blood vessels) in TC and NT tissues, and examined the intensity of PPAR-α, -β, and-γ expression. There were no significant differences in the intensity of PPAR-α, -β expression between all categories of TC and NT tissues. However, PPAR-γ expression was significantly more extensive and intense in all categories of TC than in NT tissues. Next, we demonstrated that PPAR-γ ligands induced the reduction of viability in TC cells in the range of 5–40 μM by MTT assay. Furthermore, we also demonstrated that TC cells treated with PPAR-γ ligands could induce early apoptosis and DNA fragmentation in TC cells. However, no further data on TC and PPAR-γ have been documented in other reports. We expect additional research will be progressed.

In summary, PPAR-γ expression was significantly more extensive and intense in malignant tissues than in normal tissue, and PPAR-γ expression was higher in G3 cancer than in G1 cancer. Furthermore, PPAR-γ ligands induced the reduction of malignant cell viability through apoptosis in vitro. These results indicate that PPAR-γ participates in initiation and promotion of tumorigenesis.

These results raise the possibility that PPAR-γ may play role in the pathogenesis and progression of PC and TC. While it is difficult at this time to use PPAR-γ ligands at a clinical dose (relatively nontoxic therapeutic approach) as suppressive cancer therapy, we strongly suggest that further research may confirm PPAR-γ ligands as a novel approach to the treatment of PC and TC.

## Figures and Tables

**Table 1:** Statistical analysis of PPAR-α, -β, and -γ immunostaining.

		Tumor	Blood vessel	Stromal tissue
PPAR-α	PC (N=156)			
	Low group N=50)	2.2±0.6	2.0±0.9	2.0±0.9
	Middle group N=54)	2.4±0.7	2.3±0.7	1.9±0.6
	High group N=52)	2.3±0.7	1.9±0.6	2.0±0.8
	PIN (N=15)	1.9±0.6	1.8±0.6	1.7±0.7
	BPH (N=20)	2.1±0.6	1.7±0.5	1.7±0.5
	NP (N=12)	Not present	2.1±0.7	2.2±1.1

PPAR-β	PC (N=156)			
	Low group N=50)	2.0±1.0	1.7±0.7	1.9±0.6
	Middle group N=54)	2.3±1.2	1.6±0.7	1.8±0.7
	High group N=52)	2.1±1.0	1.7±0.6	1.8±0.9
	PIN (N=15)	2.2±0.8	1.8±0.9	1.8±0.7
	BPH (N=20)	2.0±0.7	1.9±0.8	1.6±0.7
	NP (N=12)	Not present	1.9±0.7	1.9±0.8

PPAR-γ	PC (N=156)			
	Low group N=50)	2.6±0.7*	1.8±0.9*	1.8±0.7*
	Middle group N=54)	2.7±0.9*	1.8±0.8*	1.9±0.9*
	High group N=52)	3.3±1.0*	2.0±0.8*	1.7±0.9*
	PIN (N=15)	2.5±0.8*	1.9±0.8*	1.7±0.9*
	BPH (N=20)	0.8±0.6	0.6±0.5	0.7±0.5
	NP (N=12)	Not present	0.5±0.4	0.5±0.3

* Graded 0–4 on the coded sections by two blind observers. 0, no staining; 4+, maximum intensity. Statistical analysis was performed using the ANOVA (p-value). PPAR-α and -β immunostaining were significantly intense in all cases. There were no differnces among PC, PIN, BPH and NP. PPAR-γ immunostainings of tumor were significantly more extensive and intense in PC and in PIN than in tissue of BPH or NP. PPAR-γ staining was high in blood vessels and stromal tissues of PC and PIN, with no significant difference between them. However, the expressions of the PPAR-γ in the blood vessels and stromal tissues from BPH and NP were at the basic level. (* P<.01).

**Table 2:** Statistical analysis of PPAR-α, -β, and -γ immunostaining.

		Av.± SD	
	Tumor type	Epithelium	Blood vessel
PPAR-α	Seminoma (N=34)	2.2±0.8	1.7±0.9
	Embryonal carcinoma (N=15)	2.4±1.1	2.1±1.0
	Yolk sac tumor (N=11)	1.8±1.0	1.6±0.7
	Choriocarcinoma (N=10)	2.5±1.2	2.0±0.9
	Teratoma (N=12)	1.8±0.9	1.6±0.9
	Normal testis (N=20)	2.5±1.1	2.1±0.9
			
PPAR-β	Seminoma (N=34)	2.4±0.9	2.2±1.1
	Embryonal carcinoma (N=15)	2.6±1.4	2.3±1.2
	Yolk sac tumor (N=11)	2.5±1.4	2.1±0.6
	Choriocarcinoma (N=10)	2.2±1.0	1.9±0.9
	Teratoma (N=12)	2.4±0.9	2.2±1.3
	Normal testis (N=20)	2.5±1.1	2.3±1.0
			
PPAR-γ	Seminoma (N=34)	2.2±0.8*	1.9±0.9*
	Embryonal carcinoma (N=15)	2.8±1.1*	2.5±1.0*
	Yolk sac tumor (N=11)	2.2±0.9*	2.1±1.1*
	Choriocarcinoma (N=10)	2.9±1.0*	2.4±1.0*
	Teratoma (N=12)	2.0±1.3*	1.9±1.1*
	Normal testis (N=20)	0.7±0.6	0.6±0.4

* Graded 0 to 4 on the coded sections by two blind observers. 0, no staining; 4+, maximum intensity. Statistical analysis was performed using the analysis of variance (P value; ANOVA). PPAR-α, and -β immunostaining were significanty apparent in all TC and NT tissues. PPAR-γ immunostaining of tumor was significantly more extensive and intense in all TC groups than in NT tissue. PPAR-γ staining was high in blood vesseld of TC, with no significant difference between them. However, the expression of PPAR-γ in blood vessels from NT was at the basic level. P<.01.

**Table 3:** Effects of troglitazone, 15-d-PGJ2 and GW9662 in viabity of human PC and TC cell lines.

		5 μM	10 μM	20 μM	40 μM
Troglitazone					
PC cell lines	LNCaP	72.4%	25.7%	12.6%	8.4%
	PC3	48.6%	15.5%	14.7%	6.5%
	DU-145	60.1%	35.1%	7.6%	7.7%
TC cell line	NEC-8	38.7%	35.3%	36.6%	38.1%

15-d-PGJ2					
PC cell lines	LNCaP	78.9%	63.7%	22.4%	5.6%
	PC3	69.7%	59.0%	34.1%	6.8%
	DU-145	73.8%	59.3%	5.8%	5.8%
TC cell line	NEC-8	75.1%	66.7%	52.3%	46.8%

GW9662					
PC cell lines	LNCaP	106.8%	112.4%	103.7%	106.2%
	PC3	116.8%	118.6%	119.4%	120.2%
	DU-145	122.6%	119.4%	117.8%	115.6%
TC cell line	NEC-8	108.4%	115.5%	110.6%	112.3%

The dose-response analysis of viability in human cancer cells treated with troglitazone, 15-d-PGJ2 and GW9662 (5–40 μM, 48 hr) was measured by the MTT assay and expressed as % of control culture conditions (N=6).

## References

[1] Oh WK, Kantoff PW (1998). Management of hormone refractory prostate cancer: current standards and future prospects. *The Journal of Urology*.

[2] Bosari S, Lee AKC, DeLellis RA, Wiley BD, Heatley GJ, Silverman ML (1992). Microvessel quantitation and prognosis in invasive breast carcinoma. *Human Pathology*.

[3] Chinetti G, Griglio S, Antonucci M (1998). Activation of proliferator-activated receptors α and γ induces apoptosis of human monocyte-derived macrophages. *Journal of Biological Chemistry*.

[4] Spiegelman BM (1998). PPAR-γ: adipogenic regulator and thiazolidinedione receptor. *Diabetes*.

[5] Mueller E, Sarraf P, Tontonoz P (1998). Terminal differentiation of human breast cancer through PPARγ. *Molecular Cell*.

[6] Sarraf P, Mueller E, Jones D (1998). Differentiation and reversal of malignant changes in colon cancer through PPARγ. *Nature Medicine*.

[7] Tsubouchi Y, Sano H, Kawahito Y (2000). Inhibition of human lung cancer cell growth by the peroxisome proliferator-activated receptor-γ agonists through induction of apoptosis. *Biochemical and Biophysical Research Communications*.

[8] Takahashi N, Okumura T, Motomura W, Fujimoto Y, Kawabata I, Kohgo Y (1999). Activation of PPARγ inhibits cell growth and induces apoptosis in human gastric cancer cells. *FEBS Letters*.

[9] Altiok S, Xu M, Spiegelman BM (1997). PPARγ induces cell cycle withdrawal: inhibition of E2f/DP DNA-binding activity via down-regulation of PP2A. *Genes & Development*.

[10] Bishop-Bailey D, Hla T (1999). Endothelial cell apoptosis induced by the peroxisome proliferator- activated receptor (PPAR) ligand 15-deoxy-$\Delta^{12,14}$-prostaglandin ${\text{J}}_{2}$. *Journal of Biological Chemistry*.

[11] Inoue K-I, Kawahito Y, Tsubouchi Y (2001). Expression of peroxisome proliferator-activated receptor γ in renal cell carcinoma and growth inhibition by its agonists. *Biochemical and Biophysical Research Communications*.

[12] Yoshimura R, Matsuyama M, Segawa Y (2003). Expression of peroxisome proliferator-activated receptors (PPARs) in human urinary bladder carcinoma and growth inhibition by its agonists. *International Journal of Cancer*.

[13] Segawa Y, Yoshimura R, Hase T (2002). Expression of peroxisome proliferator-activated receptor (PPAR) in human prostate cancer. *The Prostate*.

[14] Hase T, Yoshimura R, Mitsuhashi M (2002). Expression of peroxisome proliferator-activated receptors in human testicular cancer and growth inhibition by its agonists. *Urology*.

[15] Yoshimura R, Matsuyama M, Hase T (2003). The effect of peroxisome proliferator-activated receptor-gamma ligand on urological cancer cells. *International Journal Of Molecular Medicine*.

[16] Paltoo D, Woodson K, Taylor P, Albanes D, Virtamo J, Tangrea J (2003). *Pro12Ala* polymorphism in the peroxisome proliferator-activated receptor-gamma (PPAR-γ) gene and risk of prostate cancer among men in a large cancer prevention study. *Cancer Letters*.

[17] Subbarayan V, Sabichi AL, Kim J (2004). Differential peroxisome proliferator-activated receptor-γ isoform expression and agonist effects in normal and malignant prostate cells. *Cancer Epidemiology Biomarkers & Prevention*.

[18] Butler R, Mitchell SH, Tindall DJ, Young CYF (2000). Nonapoptotic cell death associated with S-phase arrest of prostate cancer cells via the peroxisome proliferator-activated receptor γ ligand, 15-Deoxy-$\Delta^{12,14}$-prostaglandin ${\text{J}}_{2}$. *Cell Growth & Differentiation*.

[19] Auboeuf D, Rieusset J, Fajas L (1997). Tissue distribution and quantification of the expression of mRNAs of peroxisome proliferator-activated receptors and liver X receptor-alpha in humans: no alteration in adipose tissue of obese and NIDDM patients. *Diabetes*.

[20] Sano H, Engleka K, Mathern P (1993). Coexpression of phosphotyrosine-containing proteins, platelet-derived growth factor-B, and fibroblast growth factor-1 in situ in synovial tissues of patients with rheumatoid arthritis and Lewis rats with adjuvant or streptococcal cell wall arthritis. *The Journal of Clinical Investigation*.

[21] Kujubu DA, Reddy ST, Fletcher BS, Herschman HR (1993). Expression of the protein product of the prostaglandin synthase-2/TIS10 gene in mitogen-stimulated Swiss 3T3 cells. *Journal of Biological Chemistry*.

[22] Fitzgerald SM, Flinn S (2000). Evaluating research studies using the analysis of variance (ANOVA): issues and interpretations. *Journal of Hand Therapy*.

[23] Braissant O, Foufelle F, Scotto C, Dauça M, Wahli W (1996). Differential expression of peroxisome proliferator-activated receptors (PPARs): tissue distribution of PPAR-α, -β, and -γ in the adult rat. *Endocrinology*.

[24] Dreyer C, Krey G, Keller H, Givel F, Helftenbein G, Wahli W (1992). Control of the peroxisomal β-oxidation pathway by a novel family of nuclear hormone receptors. *Cell*.

[25] Kliewer SA, Umesono K, Noonan DJ, Heyman RA, Evans RM (1992). Convergence of 9-*cis* retinoic acid and peroxisome proliferator signalling pathways through heterodimer formation of their receptors. *Nature*.

[26] Kliewer SA, Forman BM, Blumberg B (1994). Differential expression and activation of a family of murine peroxisome proliferator-activated receptors. *Proceedings of the National Academy of Sciences of the United States of America*.

[27] Keelan JA, Sato TA, Marvin KW, Lander J, Gilmour RS, Mitchell MD (1999). 15-Deoxy-$\Delta^{12,14}$-prostaglandin ${\text{J}}_{2}$ a ligand for peroxisome proliferator-activated receptor-γ, induces apoptosis in JEG3 choriocarcinoma cells. *Biochemical and Biophysical Research Communications*.

[28] Xin X, Yang S, Kowalski J, Gerritsen ME (1999). Peroxisome proliferator-activated receptor γ ligands are potent inhibitors of angiogenesis in vitro and in vivo. *Journal of Biological Chemistry*.

[29] Boehm T, Folkman J, Browder T, O'Reilly MS (1997). Antiangiogenic therapy of experimental cancer does not induce acquired drug resistance. *Nature*.

[30] O'Reilly MS, Boehm T, Shing Y (1997). Endostatin: an endogenous inhibitor of angiogenesis and tumor growth. *Cell*.

[31] Drevs J, Hofmann I, Hugenschmidt H (2000). Effects of PTK787/ZK 222584, a specific inhibitor of vascular endothelial growth factor receptor tyrosine kinases, on primary tumor, metastasis, vessel density, and blood flow in a murine renal cell carcinoma model. *Cancer Research*.

